# Evaluating human landing catches as a measure of mosquito biting and the importance of considering additional modes of action

**DOI:** 10.1038/s41598-024-61116-0

**Published:** 2024-05-20

**Authors:** Emma L Fairbanks, Mgeni M. Tambwe, Jason Moore, Ahmed Mpelepele, Neil F. Lobo, Rajabu Mashauri, Nakul Chitnis, Sarah J. Moore

**Affiliations:** 1 Department of Epidemiology and Public Health, Swiss Tropical and Public Health Institute, Kreuzstrasse 2, Allschwill, Basel, 4123 Switzerland; 2 University of Basel, Petersplatz 1, 4001 Basel, Switzerland; 3 The Zeeman Institute for Systems Biology and Infectious Disease Epidemiology Research, Mathematics Institute, University of Warwick, Coventry, CV4 7AL UK; 4 Vector Control Product Testing Unit, Ifakara Health Institute, P.O. Box 74, Bagamoyo, Tanzania; 5 Eck Institute for Global Health, University of Notre Dame, Notre Dame, IN USA; 6 The Nelson Mandela, African Institution of Science and Technology, School of Life Sciences and Bio Engineering, Tengeru, Arusha United Republic of Tanzania

**Keywords:** Malaria, Anopheles, Volatile pyrethroid, Spatial repellent, Vector control, Entomological trials, Computational biology and bioinformatics, Diseases

## Abstract

Entomological evaluations of vector control tools often use human landing catches (HLCs) as a standard measure of a direct human-vector contact. However, some tools have additional characteristics, such as mortality, and HLCS are not sensitive for measuring other effects beyond landing inhibition. Therefore, additional measures may need to be considered when evaluating these tools for public health use. This study has two main aims (1) the evaluate the accuracy of HLCs as a proxy for feeding and (2) to compare the predicted reduction in vectorial capacity when we do and do not consider these additional characteristics. To achieve this, we analyse previously published semi-field data from an experiment which used HLCs and another where mosquitoes were allowed to feed in the presence of different dosages of the volatile pyrethroid spatial repellent, transfluthrin. We compare results for two mathematical models: one which only considers the reduction in feeding effect and one which also considers mortality before and after feeding (using data gathered by the aspiration of mosquitoes after the semi-field feeding/landing period and 24 h survival monitoring). These Bayesian hierarchical models are parameterised using Bayesian inference. We observe that, for susceptible mosquitoes, reduction in landing is underestimated by HLCs. For knockdown resistant mosquitoes the relationship is less clear; with HLCs sometimes appearing to overestimate this characteristic. We find HLCs tend to under-predict the relative reduction in vectorial capacity in susceptible mosquitoes while over-predicting this impact in knockdown-resistant mosquitoes. Models without secondary effects have lower predicted relative reductions in vectorial capacities. Overall, this study highlights the importance of considering additional characteristics to reduction in biting of volatile pyrethroid spatial repellents. We recommend that these are considered when evaluating novel vector control tools.

## Introduction

Typically, entomological evaluations of vector control tools, that reduce mosquito bites, measure exposure to vectors using a standard measure of human landing catches (HLCs)^1,2^. Here, mosquitoes are captured from the lower limbs of a human volunteer as they land, but before they bite. HLC is a direct measurement of human-vector contact that has not been reproduced accurately using traps^3–5^, but it has disadvantages especially with wild-field mosquitoes, as pathogen transmission could occur^5^. There is some experimental evidence that HLCs provide a reasonable proxy of human biting^6,7^. However, HLC measures only mosquito attack rate and is not sensitive for measuring other effects of vector control tools beyond landing inhibition^8^. There are a number of vector control tools available that reduce mosquito bites using insecticides, including volatile pyrethroid spatial repellents^9^ and pyrethroid treated clothing^10^. To estimate the potential efficacy of these kinds of interventions when applied for public health, it is relevant to measure modes of action beyond repellency including feeding inhibition, knockdown and mortality^2,11^.

Recently, semi-field studies are increasingly used to safely improve our understanding of the modes of action of vector-control tools^12^, i.e. how they affect mosquitoes’ behavioural and mortality endpoints that may be relevant when measuring the impact of interventions on vectorial capacity^13^. Here, since a known number of laboratory reared vectors are released into a cage in which they cannot escape, behavioural endpoints can be reported at the individual mosquito level. Possible endpoints include feeding, repellence, blood-feeding inhibition and mortality.

Denz et al.^14^ suggested models and parameterisation methods, based on HLC data alone, for semi-field trials aiming to characterise vector-control interventions. Here, the decrease in HLCs for a human protected by the intervention, compared to an unprotected human, was assumed to be due to preprandial mortality (death before feeding) or disarming (mosquito inhibited from blood feeding for two days). A later semi-field trial included aspiration of mosquitoes remaining the semi-field cage after the HLC collection period and examining if they were knocked down or resting. HLC collected mosquitoes and mosquitoes resting mosquitoes collected by aspiration were offered a blood meal. Fairbanks et al.^15^ used data from this study to extend the Denz et al.^14^ framework, enabling quantification of the preprandial killing and disarming effects. In this model the reduction in feeding can be attributed to combinations of preprandial mortality, disarming and repellency. Both studies also modelled and parameterised postprandial mortality, considering the probability of death after 24 hours mosquitoes. This anlysis was performed on all mosquitoes caught by HLC in Denz et al.^14^, whereas Fairbanks et al.^15^ considered mosquitoes caught by HLC which fed when offered a blood meal.

In this study we will adapt the Fairbanks et al.^15^ methodology to model and parameterise data recently published in Tambwe et al.^6^, for the volatile pyrethroid spatial repellent, transfluthrin. Here two semi-field experiments were performed with Anopheles mosquitoes; one where standard HLCs were performed and the other where mosquitoes were allowed to feed on volunteers. Here, since Tambwe et al.^6^ also included mosquito aspiration after the HLC collection period we are able to parameterise preprandial mortality and repellency. We compare results when analysing the feeding or HLC datasets for two models; one considering the potential secondary effects of preprandial and postprandial mortality using the aspiration and survival data and one that assumes the only data collected is the number of fed/HLC mosquitoes. We will compare the parameter estimates as well as how they impact the predicted reduction in vectorial capacity, based on a previous published model^16^.

## Semi-field study data

Semi-field experiments were carried out at Ifakara Health Institute. Each night included two experiments: one where mosquitoes were allowed to bite the human and one where HLCs were performed. Here we will refer to these as the feeding and landing experiments, respectively. Full details of the experimental method are in Tambwe et al.^6^. Experiments were performed for six nights per dosage (5, 10, 15 and 20g transfluthrin), with control and intervention arms performed in parallel.

**Figure 1:**
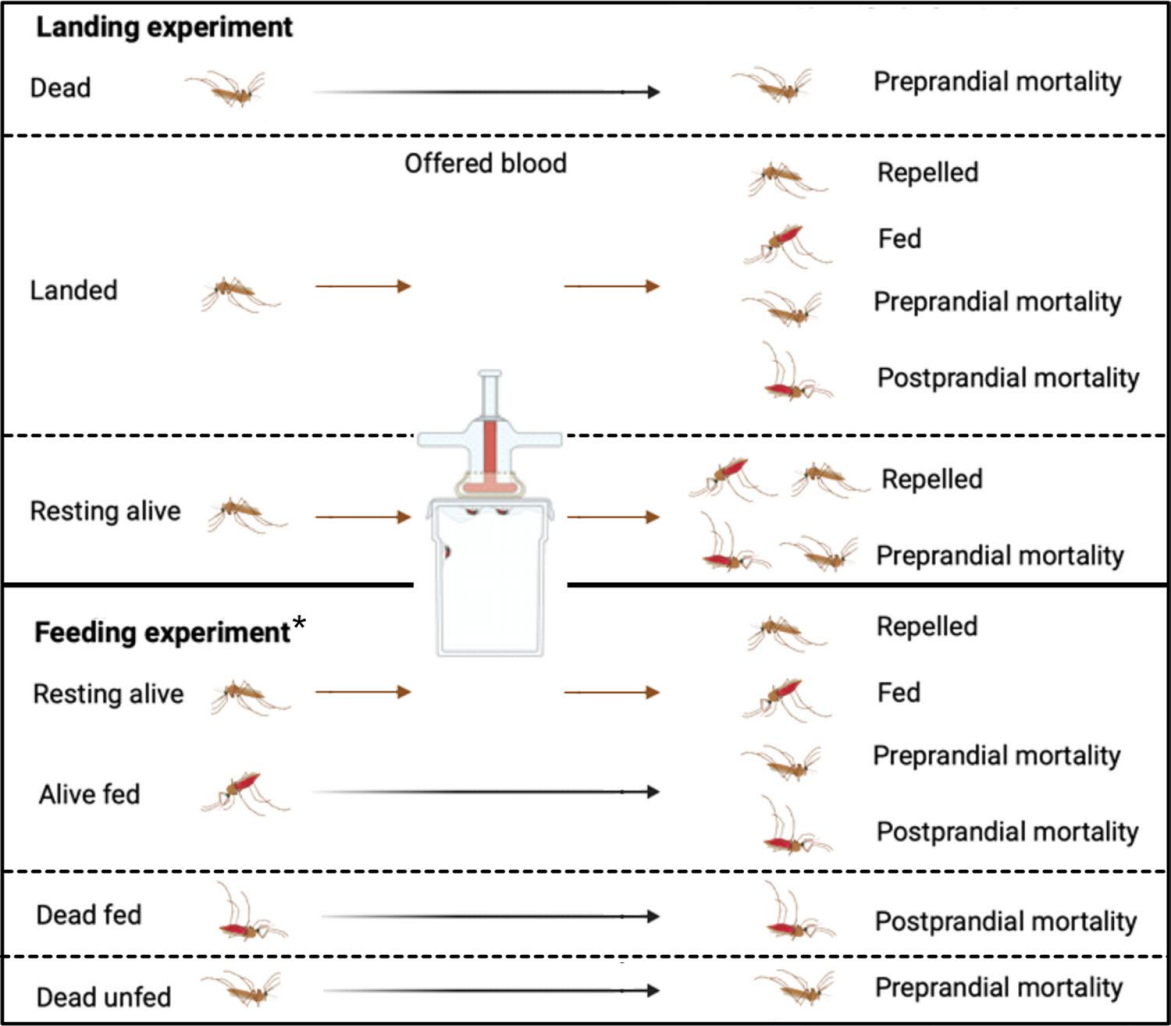
Schema showing the categorisations of mosquitoes after the semi-field-enclosure duration, whether they were offered a blood meal and the corresponding classifications after 24 hours. *Resting alive and alive fed mosquitoes were pooled for the 24-hour assessment. Figure Made in BioRender.com.

**Mosquito preparation and release** For each experiment and arm, twenty insectary-reared mosquitoes of each species—susceptible *Anopheles funestus*, susceptible *An. gambiae* sensu stricto and knockdown resistant *An. gambiae*—were realised into screen houses.

**Mosquito feeding/landing hour** For the feeding experiments volunteers allowed the mosquitoes to feed on them for one hour. For the landing experiments volunteers performed HLCs for one hour. Mosquitoes caught by HLC were placed in a collection cup. Then, in both experiments, at the end of the one-hour feeding period the mosquitoes remaining in the screen house were collected by aspiration.

**Immediate assessment** The number of knocked-down, resting and blood-fed or HLC mosquitoes were recorded for the feeding or landing experiments, respectively. HLC and resting mosquitoes were subsequently offered a blood meal for 15 minutes using the membrane technique. Following the feeding, each mosquito cup received 10% sucrose and kept under laboratory conditions of 27±2^∘^C and 75%±20% relative humidity.

**24-hour assessment** All mosquitoes were examined after 24 hours and classified as either unfed and alive, fed and alive, unfed and dead or fed and dead. Mosquitoes resting at the end of the collection hour which were dead after 24 hours were reclassified as dead. It is assumed that these mosquitoes would not have survived until the next days feeding period, and therefore would have died before feeding on a human and potentially transmitting malaria parasites. In the feeding experiment mosquitoes that fed during the feeding hour and mosquitoes found resting and offered an blood meal were pooled for the 24-hour assessment.

## Methods

In order to consider the nightly variations in the parameters, we use Bayesian hierarchical models, which incorporate hierarchical structures in the data, allowing modelling of different levels of variation in the data (Supplementary file 1).

Bayesian inference was performed using Stan^17^ in Rstudio^18^. Weakly informed priors similar to^14^ were used (Table S1). For each model parameterised, we run four Markov chains with 6000 iterations, removing the first 3000 for burn in. The convergence of chains was checked using the diagnostics available within Stan. Figures were generated using the *ggplot2* package in Rstudio^18,19^.

### Mosquito host seeking behaviour

We consider stochastic continuous-time Markov models of host-seeking behaviour of individual mosquitoes^14,16^.

#### Model with secondary effects

The mosquito starts in the host-seeking stage (*A*). Then the mosquito can either remain in *A*, feed (*B*) or be killed preprandially (*M*). We assume that the probability mosquitoes leave *A* follows an exponential distribution depending on the rates of feeding and preprandial mortality, which for an unprotected host are given as $\alpha _B$ and $\alpha _M$, respectively. These rates are assumed to have a hierarchical structure, allowing for daily variations. During a time step of duration *t* the probabilities a mosquito remains host seeking, lands/feeds or dies preprandially are given as

1 $$\begin{aligned} P_A(t) &=\exp \big ( -(\alpha _B + \alpha _M)t \big ), \end{aligned}$$

2 $$\begin{aligned} P_B(t) &=\big ( 1-P_A(t) \big ) \frac{\alpha _B}{\alpha _B + \alpha _M}, \end{aligned}$$

3 $$\begin{aligned} P_M(t) &=\big ( 1-P_A(t) \big ) \frac{\alpha _M}{\alpha _B + \alpha _M}, \end{aligned}$$

respectively. Since the rates have a hierarchical structure, these probabilities vary daily within the model. Mosquitoes are classified as host seeking (*A*), fed (*B*) or killed preprandial (*M*) and these data were fit to a multinomial distribution using the method described in Fairbanks et al.^15^.

After biting a human host mosquitoes can either survive to lay eggs and then begin host seeking again or die postprandially. Here we parameterise postprandial killing using a model defined in Denz et al.^14^. Previously, for a landing experiment, Denz et al.^14^ estimated the probability of death based on the survival of all mosquitoes caught by HLC. However, Fairbanks et al.^15^ applied this model to only mosquitoes caught by HLC that fed when offered a blood meal. For the landing experiment, we compare results from applying the model to these two datasets. For the feeding experiment, the mosquitoes that fed in the semi-field chamber and mosquitoes found resting which fed when offered a blood meal were pooled for the 24-hour survival analysis. Therefore in the feeding experiment, we cannot distinguish between postprandial mortality of mosquitoes that fed in the semi-field chamber or those that were resting in the sem-field chamber and only fed when offered a blood meal subsequently. We assume that mosquitoes fed and alive at 24 hours were biting mosquitoes. If there were fewer fed alive mosquitoes at 24 hours, compared to after the initial evaluation immediately after the feeding-period, we assume this was due to postprandial killing. We fit a binomial distribution to estimate the probability of postprandial mortality, $P_S$, which has a hierarchical structure^14^.

#### Model without secondary effects

In this model there is no preprandial mortality. Therefore, the mosquito can either remain in *A* or feed (*B*). For this model during a time step of duration *t* the probabilities a mosquito remains host seeking or lands/feeds are given as

4 $$\begin{aligned} P_A(t) &=\exp \big ( -\alpha _B t \big ) \text { or} \end{aligned}$$

5 $$\begin{aligned} P_B(t) &=\big ( 1-P_A(t) \big ), \end{aligned}$$

respectively.

Here we assume only data on the number of HLCs or fed mosquitoes is collected, which we fit to a binomial distribution. The probability of postprandial mortality is modelled and fit using the same methods as the model with secondary effects.

### Intervention effects

We denote rates of biting and preprandial mortality and the postprandial mortality probability for a unprotected host as $\bar{\alpha }_B$, $\bar{\alpha }_M$ and $\bar{P}_S$, respectively. These are then adjusted for a host protected by the intervention. The adjusted rate of landing/feeding, often referred to as the host-availability rate, is

6 $$\begin{aligned} \hat{\alpha }_B = (1 - \pi ) \bar{\alpha }_B, \end{aligned}$$

where $0 \le \pi \le 1$ is the reduction in the host-availability rate for mosquitoes encountering a human protected by the intervention compared to an unprotected (control) human. The preprandial mortality rate for a protected host is

7 $$\begin{aligned} \hat{\alpha }_M = \bar{\alpha }_M + \kappa \bar{\alpha }_B, \end{aligned}$$

where $0 \le \kappa$ is the increase in the rate of preprandial mortality due to an intervention relative to the host-availability rate of an unprotected human (i.e. the rate mosquitoes die preprandially instead of biting). This is only estimated for the model with secondary effects, since there is no $\alpha _M$ in the model without secondary effects.

Given the probability of a mosquito dying postprandially after biting an unprotected human, $\bar{P}_S$, we assume the probability of postprandial mortality for a mosquito which feeds on a human protected an intervention is

8 $$\begin{aligned} \hat{P}_S = \bar{P}_S + (1 - \bar{P}_S) \xi , \end{aligned}$$

where $0 \le \xi \le 1$ is the increased probability of mortality. This is only considered in the model with secondary effects.

### Reduction in vectorial capacity

Vectorial capacity, defined as the total number of potentially infectious bites that would eventually arise from all the mosquitoes biting a single perfectly infectious human on a single day^20^, is a measure of the ability of the vector population to transmit a disease. The availability rates of humans and non-human hosts are estimated using a method described in Briët et al.^21^, scaling the relative attractiveness according to the human blood index. Using these rates, along with estimated parameters, a previously published model is used to determine the relative reduction in vectorial capacity when an intervention is utilised with a range of *coverage levels* within the population^16^. Here coverage refers to the percentage of intervention use within the population. The model assumes that vectorial capacity is proportional to mosquito emergence rate and is not affected by the larval carrying capacity. To show the potential impact of assuming HLCs are a proxy for mosquito biting and not considering other modes of action, model outputs from the estimates from the feeding and landing experiments, and with and without secondary effects are compared.

Entomological parameters for calculating the vectorial capacity in a setting without interventions (all human hosts unprotected) are given in Table **S2**. The parameters for the non-human hosts and unprotected hosts (humans without the tools) remain the same in the intervention scenarios. For the intervention scenarios, a proportion of human hosts are protected by an intervention — these hosts have a rate of biting which is reduced by $\pi$. When considering secondary effects postprandial killing is included by scaling the probability a mosquito finds a resting place by $(1 - \xi )$, compared to the probability of an unprotected host. An additional ’dummy’ host, which cannot transmit malaria, is used to include the effects of preprandial mortality. One dummy host is generated for each host using the intervention, with an availability rate of the dummy host equal to that of an unprotected human scaled by $\kappa$, and all mosquitoes that interact with this host are killed.

## Results

### Intervention effects parameter estimates

**Figure 2:**
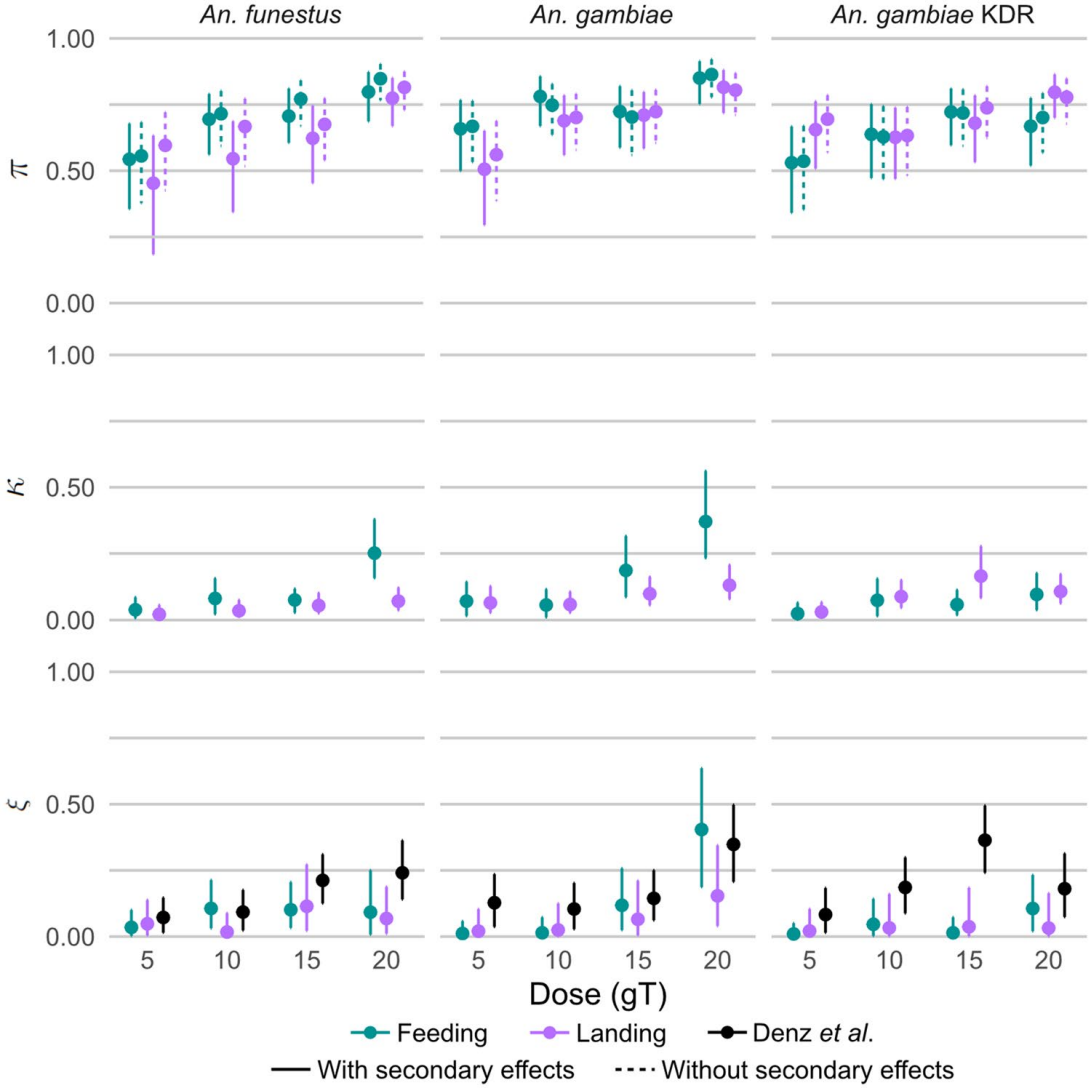
Median parameter estimates and 95% confidence intervals for the reduction in the host-availability rate for mosquitoes encountering a human protected by the intervention ($\pi$), the increase in the rate of preprandial mortality due to an intervention ($\kappa$) relative to the host-availability rate of an unprotected human and the increase in the probability of postprandial mortality for a mosquito which feeds on a human protected an intervention ($\xi$). For $\xi$, the landing result refers to the Fairbanks et al.^15^ method.

Figure 2 shows the differences in $\pi$ and $\kappa$ estimates for the feeding and landing experiments, for the models with and without secondary effects, for each dosage and species. We observe that, for the susceptible mosquitoes, $\pi$ is underestimated in the landing experiment. For the largest dose, the feeding experiments have larger estimates of $\kappa$. A possible explanation for this is mosquitoes may land on a protected human, but not bite, and later die from their interaction with the intervention. In the biting experiment this behaviour would be classified as preprandial mortality, however in the landing experiment the mosquito would have been caught by HLC.

For the knockdown resistant mosquitoes the relationship is less clear; with HLCs appearing to overestimate $\pi$ for the smallest and largest dose. For the experiment with 15 grams of transfluthrin knockdown-resistant mosquitoes landing appears to overestimate $\kappa$, however this is not observed for other dosages.

When observing confidence intervals (CIs) for the parameters derived from the landing datasets, $\pi$ CIs appear overall larger for the model with secondary effects. This pattern is not observed for parameters derived from the feeding experiments.

Figure 2 shows the estimates for the postprandial killing effect, $\xi$. Here it is important to note that due to assuming fed and alive mosquitoes at 24 hours were biting mosquitoes, rather then resting mosquitoes that fed when offered a blood meal in the feeding experiments, the postprandial killing effect in the feeding model may be underestimated. We observe that for the landing experiment $\xi$ estimations are larger considering all HLC caught mosquitoes (^14^) compared to only HLC mosquitoes which feed (Fairbanks et al.^15^). In most experiments the dataset which only considers HLC mosquitoes which feed gives estimates closer to the dataset considering all HLC mosquitoes.

### Relative reduction in vectorial capacity predictions

**Figure 3:**
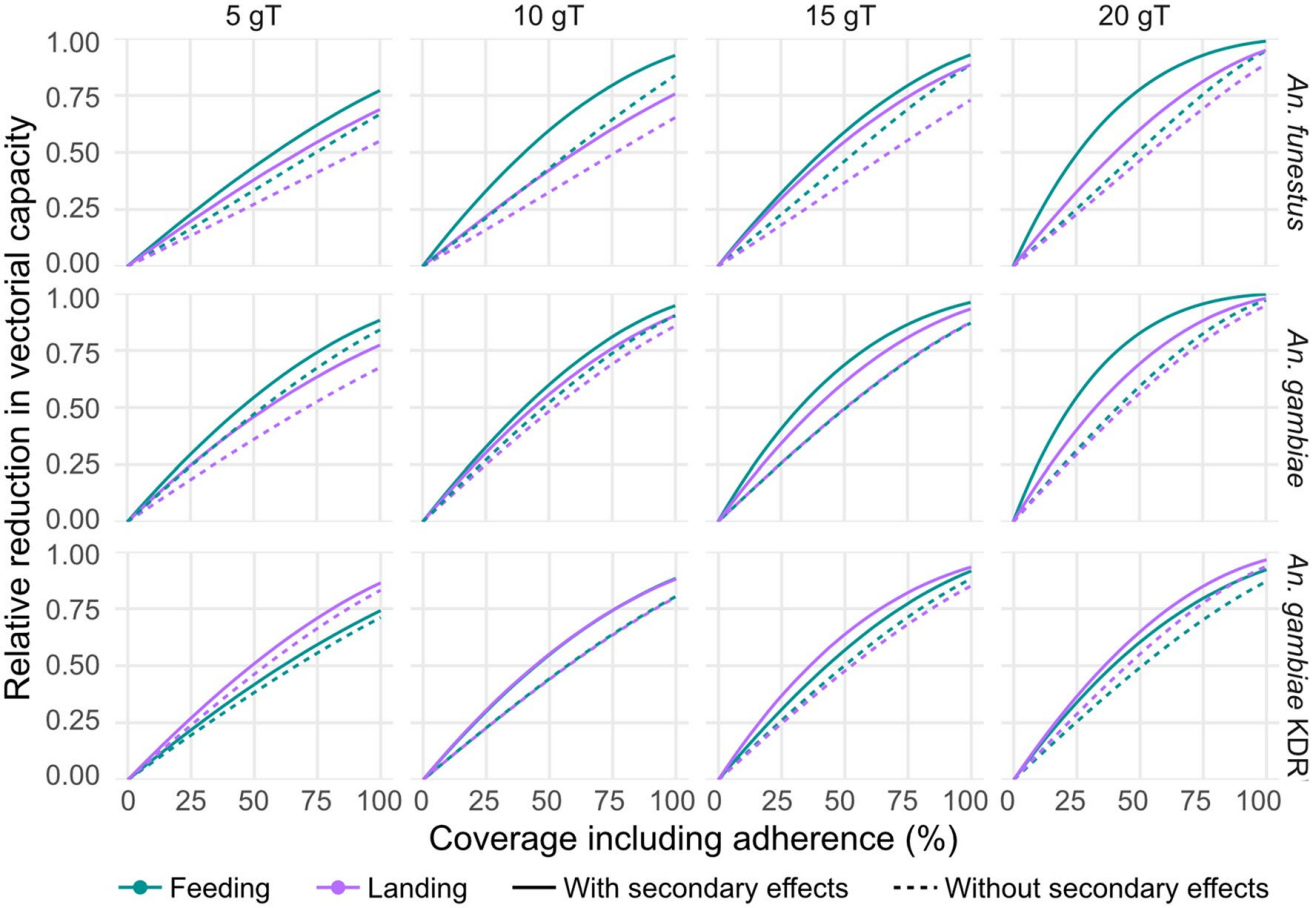
The predicted relative reduction in vectorial capacity with parameters derived from the models with and without secondary effects for each experiment.

Figure 3 shows the predicted relative reduction in vectorial capacity for each model, experiment, species and dosage. We observe that HLCs tend to under-predict the relative reduction in vectorial capacity in susceptible mosquitoes while over-predicting this impact in knockdown resistant mosquitoes, especially with the smallest and largest dosage.

Parameter estimates derived from the models without secondary effects have lower predicted relative reductions in vectorial capacities. This is particularly the case for lower coverage levels. The difference in the predictions is often more for parameters derived from the feeding experiment, compared to the landing experiment.

## Discussion

This study shows that not considering secondary effects of vector bite prevention tools, such as preprandial and postprandial killing, does not fully assess their potential impact. Rather, reduction in biting is just one of many modes of action which interrupt malaria transmission in mosquitoes. Using mathematical modelling, we were able to quantify the difference of a hypothetical volatile pyrethroid based bite prevention tool’s impact on the ability of mosquitoes to transmit malaria when these secondary effects are considered, which were much greater than when only the reduction in biting is considered.

We also show that estimates based on feeding and landing data have discrepancies. For larger dosages of transfluthrin, more preprandial killing was observed in the feeding experiments. This is presumably because mosquitoes will spend more time in close contact to the host while locating a suitable site to bite, especially if their olfactory system has been disrupted, whereas the landing experiment mosquitoes were removed as soon as they landed on a host and placed into a paper cup, where they may have been less exposed to insecticide^22,23^. For postprandial mortality the estimates from the feeding and landing experiments more closely align when considering only mosquitoes that fed when offered a blood meal in the landing experiment.

A limitation of this work is that, in the feeding experiment, mosquitoes which were collected as fed and resting were pooled. It was assumed that fed alive mosquitoes at 24 hours after aspiration were mosquitoes that fed during the feeding-period in the semi-field chamber when estimating the postprandial killing for the feeding experiments. Alternatively, some of these mosquitoes may have been resting, fed when offered a blood meal and then died within 24 hours. If this was the case, postprandial killing would be under-estimated. We chose to make this more conservative assumption, rather than allow for mortality to be overestimated. Future experiments should separate those mosquitoes which bite the host (found resting fed) and unfed resting mosquitoes that feed after recapture. This would allow for more accurate classifications of mosquitoes as repelled, preprandially killed and postprandially killed (Fig. 1). This study used data from 120 mosquitoes of each study, released in batches of 20 over 6 nights. Using data with more mosquitoes released each night, over more nights, would allow for more certainty in results. Overall, this study highlights the importance of secondary effects, additional to reduction in biting, of volatile pyrethroid spatial repellents.

Semi-field studies allow for more detailed analysis on multiple mosquito endpoints, compared to field trials. This gives insights into the reasons for observed reductions in biting. However, reductions in landing observed in semi-field environments may not represent the true reduction of biting. Volatile pyrethroids diffuse airborne concentrations of an active ingredients. Therefore, since semi-field studies are performed inside an enclosed environment, concentration may be higher in this environment, compared to the outside, with environmental factors, such as wind speed, possibly reducing concentrations of these active ingredients^24^. However, a recent field study observed a significant reduction in landing in temporary shelters with volatile-pyrethroid spatial emanators^25^. Community protection of transfluthrin-treated eave ribbons has been observed through reductions in landing amongst unprotected individuals in experimental huts^26^.

Recent epidemiological trials for an transfluthrin-based passive emanator have demonstrated reductions in malaria^27^. It is likely that the clinical impact of such interventions against malaria is greater than that measured via traditional mosquito endpoints such as indoor density or human landings^7^. Evaluation of volatile pyrethroids is best conducted using assays that can capture all modes of action including semi-field studies or experimental hut trials^28^. Furthermore, in a community setting, measures of impact of the intervention on mosquito population level endpoints such as sporozoite rate or population age may be more appropriate^29^. There may be additional modes of action beyond blood feeding behaviour, such as fecundity, which could also impact disease transmission^30^.

This study demonstrated that the mode of action of transfluthrin applied as a spatial repellent goes beyond bite prevention. It is important to consider secondary effects of tools, such as preprandial and postprandial killing, especially when considering the use of volatile pyrethroids for public health applications for disease prevention and control^31^. The model demonstrated likely community level impacts when volatile pyrethroids are deployed at scale.

### Supplementary Information

Supplementary Information.

## Data Availability

The datasets analysed during the current study are published in^6^ available at https://doi.org/10.1186/s13071-023-05685-5. Stan codes used for data analysis are available in the Supplementary Information files.

## References

[1] Harrington LC, Foy BD, Bangs MJ (2020). Considerations for human blood-feeding and arthropod exposure in vector biology research: An essential tool for investigations and disease control. Vector Borne Zoonotic Dis..

[2] World Health Organization and others. Guidelines for efficacy testing of mosquito repellents for human skin. Technical report, World Health Organization (2009a). https://iris.who.int/bitstream/handle/10665/70072/WHO_HTM_NTD_WHOPES_2009.4_eng.pdf?sequence=1&isAllowed=y.

[3] Yan C, Hii J, Ngoen-Klan R, Saeung M, Chareonviriyaphap T (2022). Semi-field evaluation of human landing catches versus human double net trap for estimating human biting rate of Anopheles minimus and Anopheles harrisoni in Thailand. PeerJ.

[4] Namango IH, Marshall C, Saddler A, Ross A, Kaftan D, Tenywa F, Makungwa N, Odufuwa OG, Ligema G, Ngonyani H, Matanila I, Bharmal J, Moore J, Moore SJ, Hetzel MW (2022). The Centres for Disease Control light trap (CDC-LT) and the human decoy trap (HDT) compared to the human landing catch (HLC) for measuring Anopheles biting in rural Tanzania. Malar. J..

[5] Gao Q, Wang F, Lv X, Cao H, Zhou J, Su F, Xiong C, Leng P (2018). Comparison of the human-baited double net trap with the human landing catch for Aedes albopictus monitoring in Shanghai. China. Parasit. Vectors.

[6] Tambwe MM, Kibondo UA, Odufuwa OG, Moore J, Mpelepele A, Mashauri R, Saddler A, Moore SJ (2023). Human landing catches provide a useful measure of protective efficacy for the evaluation of volatile pyrethroid spatial repellents. Parasit. Vectors.

[7] Swai JK, Kibondo UA, Ntabaliba WS, Ngoyani HA, Makungwa NO, Mseka AP, Chura MR, Mascari TM, Moore SJ (2023). CDC light traps underestimate the protective efficacy of an indoor spatial repellent against bites from wild Anopheles arabiensis mosquitoes in Tanzania. Malar. J..

[8] Bibbs CS, Kaufman PE (2017). Volatile pyrethroids as a potential mosquito abatement tool: a review of pyrethroid-containing spatial repellents. J. Integr. Pest Manag..

[9] Ogoma SB, Moore SJ, Maia MF (2012). A systematic review of mosquito coils and passive emanators: defining recommendations for spatial repellency testing methodologies. Parasit. Vectors.

[10] Banks SD, Murray N, Wilder-Smith A, Logan JG (2014). Insecticide-treated clothes for the control of vector-borne diseases: a review on effectiveness and safety. Med. Vet. Entomol..

[11] World Health Organization and others. Guidelines for efficacy testing of household insecticide products: mosquito coils, vaporizer mats, liquid vaporizers, ambient emanators and aerosols. Technical report, World Health Organization (2009b). https://iris.who.int/bitstream/handle/10665/70071/WHO_HTM_NTD_WHOPES_2009.3_eng.pdf?sequence=1.

[12] Innovative Vector Control Consortium. An Expert Review of Spatial Repellents for Mosquito Control. Technical report, Innovative Vector Control Consortium (2020). https://www.ivcc.com/wp-content/uploads/2020/08/An-Expert-Review-of-Spatial-Repellents-for-Mosquito-Control.pdf.

[13] Vontas J, Moore S, Kleinschmidt I, Ranson H, Lindsay S, Lengeler C, Hamon N, McLean T, Hemingway J (2014). Framework for rapid assessment and adoption of new vector control tools. Trends Parasitol..

[14] Denz A, Njoroge MM, Tambwe MM, Champagne C, Okumu F, van Loon JJA, Hiscox A, Saddler A, Fillinger U, Moore SJ, Chitnis N (2021). Predicting the impact of outdoor vector control interventions on malaria transmission intensity from semi-field studies. Parasit. Vectors.

[15] Fairbanks EL, Saeung M, Pongsiri A, Vajda E, Wang Y, McIver DJ, Richardson JH, Tatarsky A, Lobo NF, Moore SJ, Ponlawat A, Chareonviriyaphap T, Ross A, Chitnis N (2024). Inference for entomological semi-field experiments: Fitting a mathematical model assessing personal and community protection of vector-control interventions. Comput. Biol. Med..

[16] Chitnis N, Smith T, Steketee R (2008). A mathematical model for the dynamics of malaria in mosquitoes feeding on a heterogeneous host population. J. Biol. Dyn..

[17] Stan. *Stan user’s guide version 2.34*, (2023). https://mc-stan.org/docs/stan-users-guide/index.html.

[18] RStudio Team. *Rstudio: Integrated Development Environment for R*. RStudio, PBC., Boston, MA, (2020). URL http://www.rstudio.com/.

[19] Wickham, H., Chang, W., Henry, L., Pedersen, TL., Takahashi, K., Wilke, C., Woo, K., Yutani, H., Dunnington, D. & RStudio. *Create Elegant Data Visualisations Using the Grammar of Graphics* (2022). https://ggplot2.tidyverse.org.

[20] Garrett-Jones C (1964). The human blood index of malaria vectors in relation to epidemiological assessment. Bull. World Health Organ..

[21] Briët OJT, Impoinvil DE, Chitnis N, Pothin E, Lemoine JF, Frederic J, Smith TA (2019). Models of effectiveness of interventions against malaria transmitted by Anopheles albimanus. Malar. J..

[22] Andreazza F, Valbon W, Dong K (2023). Transfluthrin enhances odorant receptor-mediated spatial repellency in Aedes aegypti. Pestic. Biochem. Physiol..

[23] Valbon W, Andreazza F, Oliveira EE, Liu F, Feng B, Hall M, Klimavicz J, Coats JR, Dong K (2022). Bioallethrin activates specific olfactory sensory neurons and elicits spatial repellency in Aedes aegypti. Pest Manag. Sci..

[24] Kawada, H., Iwasaki, T., Luu, LL., Tran, KT., Nguyen, TNM., Shono, Y., Katayama, Y. & Takagi, M. Field evaluation of spatial repellency of metofluthrin-impregnated latticework plastic strips against *Aedes aegypti* (L.) and analysis of environmental factors affecting its efficacy in My Tho City, Tien Giang, Vietnam. *Am J Trop Med Hyg*, 25 (6): 1153–1157 (2006). 17172385

[25] Vajda, E. A., Ross, A., Doum, D., Fairbanks, E. L., Chitnis, N., Hii, J., Moore, S. J., Richardson, J., Macdonald, M., Sovannaroth, S., Kimheng, P., McIver, D. J., Tatarsky, A. & Lobo, N. F. Field evaluation of a volatile pyrethroid spatial repellent and etofenprox-treated clothing for outdoor protection against forest malaria vectors in Cambodia. *bioRvix* (2024). 10.1101/2024.01.30.577940. 10.1038/s41598-024-67470-3 PMC11284218 39069597

[26] Mwanga EP, Mmbando AS, Mrosso PC, Stica C, Mapua SA, Finda MF, Kifungo K, Kafwenji A, Monroe AC, Ogoma SB, Ngowo HS, Okumu FO (2019). Eave ribbons treated with transfluthrin can protect both users and non-users against malaria vectors. Malar. J..

[27] World Health Organization and others. Eighteenth meeting of the WHO Vector Control Advisory Group: meeting report, 24–26 April 2023 (2023). https://www.who.int/publications/i/item/9789240077300.

[28] Swai, J. K., Soto, A. C., Ntabaliba, W. S., Kibondo, U., Ngonyani, H. A., Mseka, A. P., Ortiz, A., Chura, MR., Mascari, TM. & Moore, SJ. Efficacy of the spatial repellent product Mosquito Shield,^TM^ against wild pyrethroid-resistant *Anopheles arabiensis* in south-eastern Tanzania. *Malar J.*, **22**(1), 249 (2023b). 10.1186/s12936-023-04674-4. 10.1186/s12936-023-04674-4 PMC10466708 37649032

[29] Magesa, SM., Wilkes, TJ., Mnzava, AEP, Njunwa, KJ., Myamba, J., Kivuyo, MDP, Hill,N., Lines, JD. & Curtis, CF. Trial of pyrethroid impregnated bednets in an area of Tanzania holoendemic for malaria Part 2. Effects on the malaria vector population. *Acta Trop.*, **49**(2): 97–108 (1991). 10.1016/0001-706x(91)90057-q. 10.1016/0001-706x(91)90057-q 1680284

[30] Ahebwa A, Hii J, Neoh KB, Leepasert T, Chareonviriyaphap T (2024). Effects of transfluthrin-treated jute and cotton clothing against resistant and susceptible Aedes aegypti (Diptera: Culicidae) in a semifield system. J. Med. Entomol..

[31] Achee NL, Perkins TA, Moore SM, Liu F, Sagara I, Van Hulle S, Ochomo EO, Gimnig JE, Tissera HA, Harvey SA, Monroe A, Morrison AC, Scott TW, Reiner RC, Grieco JP (2023). Spatial repellents: The current roadmap to global recommendation of spatial repellents for public health use. Curr. Res. Parasitol. Vector Borne Dis..

