# Supplementary material for: Evaluating human landing catches as a measure of mosquito biting and the importance of considering additional modes of action
Source: Sci Rep. 2024 May 20;14:11476. doi: 10.1038/s41598-024-61116-0 (PMC11106315; doi:10.1038/s41598-024-61116-0)
Supplement: Supplementary file 1 — Supplementary Information. [file 41598_2024_61116_MOESM1_ESM.pdf]

# Supplementary material: Evaluating human landing catches as a measure of mosquito biting and the importance of considering additional modes of action

Emma L Fairbanks[1,2,3,*], Mgeni M Tambwe[1,2,4], Jason Moore[1,2,4], Ahmed Mpelepele[4], Neil F Lobo[5], Rajabu Mashauri[4], Nakul Chitnis[1,2], and Sarah J Moore[1,2,4,6]

[1]Department of Epidemiology and Public Health, Swiss Tropical Public Health, Institute, Kreuzstrasse 2, 4123 Allschwill, Basel, Switzerland
[2]University of Basel, Petersplatz 1, 4001, Basel, Switzerland
[3]The Zeeman Institute for Systems Biology and Infectious Disease Epidemiology Research, Mathematics Institute, University of Warwick, Coventry, CV4 7AL, United Kingdom
[4]Vector Control Product Testing Unit, Ifakara Health Institute, P.O. Box 74, Bagamoyo, Tanzania
[5]Eck Institute for Global Health, University of Notre Dame, Notre Dame, IN, United States of America
[6]The Nelson Mandela, African Institution of Science and Technology, School of Life Sciences and Bio Engineering, Tengeru, Arusha, United Republic of Tanzania
[*]Corresponding author: emma-louise.fairbanks@warwick.ac.uk

## 1 Bayesian hierarchical models

We use Bayesian hierarchical models to consider the nightly variations in the parameters. For $\alpha_B$ and $\alpha_M$, the nightly rates for the control experiments are assumed to follow lognormal distributions. We consider $\phi_a$ and $\phi_b$, describing how $a$ and $b$ deviate nightly from the mean of of $\log(\bar{\alpha}_B)$ and $\log(\bar{\alpha}_M)$ according to the scale of the standard deviation. We denote the nightly parameter values with a subscript $k$, for $k = 1, 2, \ldots, 6$ elements, therefore the nightly rates are given as

$$\bar{\alpha}_{B,k} = \exp(a + \phi_{a,k}\sigma_a) \text{ and}$$
$$\bar{\alpha}_{M,k} = \exp(b + \phi_{b,k}\sigma_b).$$

The expected value of these distributions, i.e. the mean of the rates, are therefore calculated as

$$\bar{\alpha}_B = [\bar{\alpha}_{B,k}] = \exp(a + \sigma_a^2/2) \quad (1)$$
$$\text{and } \bar{\alpha}_M = [\bar{\alpha}_{M,k}] = \exp(b + \sigma_b^2/2). \quad (2)$$

The probability a mosquito encountering an unprotected host dies after biting ($\bar{P}_S$) is assumed to follow a logistic distribution. The nightly probability is given as

$$\bar{P}_S = \text{logit}^{-1}(z + \phi_{z,k}\sigma_z);$$

where $z$, $\sigma_z$ and $\phi_{z,k}$ are the mean, standard deviation and nightly deviation from the mean of $\text{logit}(\bar{P}_{S_k})$ on night $k$. The mean probability over all nights is calculated as

$$\bar{P}_S = [\bar{P}_{S_k}] = \text{logit}^{-1}(z). \quad (3)$$

## 2 Supplementary table S1

Table S1: Hyperparamters of the models. $k$ denotes the night for parameters that vary nightly.

| Parameter | Definition | Prior |
|---|---|---|
| $a$ | $\log(\bar{\alpha}_{B,k})$ mean | $\mathcal{N}(0, 6)$ |
| $\sigma_a$ | $\log(\bar{\alpha}_{B,k})$ standard deviation | Half-Cauchy(0,1) |
| $\phi_{a,k}$ | Normalised deviation of $\log(\bar{\alpha}_{B,K})$ from mean | $\mathcal{N}(0, 1)$ |
| $b$ | $\log(\bar{\alpha}_{M,k})$ mean | $\mathcal{N}(0, 6)$ |
| $\sigma_b$ | $\log(\bar{\alpha}_{M,k})$ standard deviation | Half-Cauchy(0,1) |
| $\phi_{b,k}$ | Normalised deviation of $\log(\bar{\alpha}_{M,K})$ from mean | $\mathcal{N}(0, 1)$ |
| $z$ | $\text{logit}(\bar{P}_{S,k})$ mean | Logistic(0,1) |
| $\sigma_z$ | $\text{logit}(\bar{P}_{S,k})$ standard deviation | Half-Cauchy(0,1) |
| $\phi_{r_k}$ | Normalised deviation of $\text{logit}(\bar{P}_{S,k})$ from mean | $\mathcal{N}(0, 1)$ |
| $1 - \pi$ | Intervention effect on host-availability rate | Lognormal(0,5) |
| $\kappa$ | Intervention effect on preprandal mortality | Lognormal(0,5) |
| $\xi$ | Intervention effect on postprandial mortality | Uniform(0,1) |

# 3 Vectorial capacity model parameters

Parameters for an unprotected host are given in Table S2.

In the original model there is a distinction between the rate of mortality while host-seeking ($\mu_{va}$) and the probability of preprandial mortality ($P_b$). In the current model, preprandial mortality is modelled as a rate, similar to mortality while host-seeking, in the original model. This technique was first adopted by [1] where, simularly to this study, a dummy host was added to model the rate of preprandial mortality. This makes results easier to interpret than the original model, which allowed for negative repellency in the presence of secondary effects. This formulation has since also been utilised in Fairbanks et al. [2] and Wang et al. [3].

Table S2: Definitions and values of parameters used to calculate the vectorial capacity. Baseline values, considering all humans as unprotected, for *Plasmodium falciparum* malaria and *Anopheles Minimus* are given.

| Symbol | Definition | *An. gambiae* | *An. funestus* | Ref. |
|---|---|---|---|---|
| $N_H$ | Number of humans | 10,000 | 10,000 | Assumption |
| $N_A$ | Number of non-human hosts | 10,000 | 10,000 | Assumption |
| $\theta_d$ | Maximum time a mosquito unsuccessfully searches for a blood meal per day | 0.33 days | 0.33 days | [4] |
| $P_B$ | Probability that a mosquito bites after encountering a host | 0.95 | 0.95 | [4] |
| $P_C$ | Probability that a mosquito finds a resting place | 0.95 | 0.95 | [4] |
| $P_D$ | Probability that a mosquito survives the resting phase | 0.99 | 0.99 | [4] |
| $P_E$ | Probability that a mosquito lays eggs and returns to host-seeking | 0.88 | 0.88 | [4] |
| $\tau$ | Time between feeding and laying eggs | 3 days | 3 days | [4] |
| $\theta_s$ | Duration of the extrinsic incubation period (time required for sporozoites to develop in the mosquito) | 11 days | 11 days | [4] |
| $A_0$ | The sac rate of mosquitoes (estimated proportion of host-seeking mosquitoes which laid eggs the same day) | 0.45 | 0.59 | [7] |
| $M$ | The parity rate of mosquitoes (proportion of host-seeking mosquitoes that have previously laid eggs) | 60.70 | 68.65 | [5] |
| $\chi$ | Human blood index (proportion of blood-fed mosquitoes which fed on a human) | 0.65 | 0.70 | [6] |
| $\alpha_{human}$ | Human availability rate | $9.02\times10^{-5}$ days$^{-1}$ | $1.64\times10^{-4}$ days$^{-1}$ | Calculated |
| $\alpha_{non-human}$ | Non-human availability rate | $4.90\times10^{-5}$ days$^{-1}$ | $7.03\times10^{-5}$ days$^{-1}$ | Calculated |
| $\mu_{vA}$ | Per-capita mosquito death rate while searching for a blood meal | 0.40 days$^{-1}$ | 0.33 days$^{-1}$ | Calculated |

# References

[1] A Denz, MM Njoroge, MM Tambwe, C Champagne, F Okumu, JJA van Loon, A Hiscox, A Saddler, U Fillinger, SJ Moore, and N Chitnis. Predicting the impact of outdoor vector control interventions on malaria transmission intensity from semi-field studies. *Parasit vectors*, 14:64, 2021. doi: 10.1186/s13071-020-04560-x.

[2] EL Fairbanks, M Saeung, A Pongsiri, E Vajda, Y Wang, DJ McIver, JH Richardson, A Tatarsky, NF Lobo, SJ Moore, A Ponlawat, T Chareonviriyaphap, A Ross, and N Chitnis. Inference for entomological semi-field experiments: Fitting a mathematical model assessing personal and community protection of vector-control interventions. *Comput Biol Med*, 168:107716, 2024. doi: 10.1016/j.compbiomed.2023.107716.

[3] Y Wang, N Chitnis, and EL Fairbanks. Optimizing malaria vector control: A systematic review and mathematical modelling study to identify desirable characteristics of novel tools in different settings. *Research Square*, 2023. doi: 10.21203/rs.3.rs-3332552/v1.

[4] OJT Briët, DE Impoinvil, N Chitnis, E Pothin, JF Lemoine, J Frederic, and TA Smith. Models of effectiveness of interventions against malaria transmitted by *Anopheles albimanus*. *Malar J*, 18:263, 2019. doi: 10.1186/s12936-019-2899-3.

[5] SI Hay, ME Sinka, RM Okara, CW Kabaria, PM Mbithi, CC Tago, D Benz, PW Gething, RE Howes, AP Patil, WH Temperley, MJ Bangs, T Chareonviriyaphap, IRF Elyazar, RE Harbach, J Hemingway, S Manguin, CM Mbogo, Y Rubio-Palis, and CJ Godfray. Developing global maps of the dominant *Anopheles* vectors of human malaria. *PLoS med*, 7(2):e1000209, 2010. doi: 10.1371/journal.pmed.1000209.

[6] J Orsborne, L Furuya-Kanamori, CL Jeffries, M Kristan, AR Mohammed, YA Afrane, K O'Reilly, E Massad, C Drakeley, T Walker, and L Yakob. Using the human blood index to investigate host biting plasticity: a systematic review and meta-regression of the three major African malaria vectors. *Malar J*, 17:479, 2018. doi: 10.1186/s12936-018-2632-7.

[7] L Kamber, EL Fairbanks, A Cavelan, MA Penny, and N Chitnis. Modelling the effectiveness of attractive targeted sugar baits in reducing clinical malaria. *BioRxiv*, 2024.
